# Fabrication of a SnO_2_-Based Acetone Gas Sensor Enhanced by Molecular Imprinting

**DOI:** 10.3390/s150100352

**Published:** 2014-12-26

**Authors:** Wenhu Tan, Xiaofan Ruan, Qiuxiang Yu, Zetai Yu, Xintang Huang

**Affiliations:** Institute of Nanoscience and Nanotechnology, Central China Normal University, Wuhan 430079, China; E-Mails: twhtiger@phy.ccnu.edu.cn (W.T.); rxf.sky@163.com (X.R.); qiuxiangyu@mails.ccnu.edu.cn (Q.Y.); ztyu@phy.ccnu.edu.cn (Z.Y.)

**Keywords:** SnO_2_, molecular imprinting, acetone gas sensor, clusters, ^17^O-NMR

## Abstract

This work presents a new route to design a highly sensitive SnO_2_–based sensor for acetone gas enhanced by the molecular imprinting technique. Unassisted and acetone-assisted thermal synthesis methods are used to synthesis SnO_2_ nanomaterials. The prepared SnO_2_ nanomaterials have been characterized by X-ray powder diffraction, scanning electron microscopy and N_2_ adsorption−desorption. Four types of SnO_2_ films were obtained by mixing pure deionized water and liquid acetone with the two types of as-prepared powders, respectively. The acetone gas sensing properties of sensors coated by these films were evaluated. Testing results reveal that the sensor coated by the film fabricated by mixing liquid acetone with the SnO_2_ nanomaterial synthesized by the acetone-assisted thermal method exhibits the best acetone gas sensing performance. The sensor is optimized for the smooth adsorption and desorption of acetone gas thanks to the participation of acetone both in the procedure of synthesis of the SnO_2_ nanomaterial and the device fabrication, which results in a distinct response–recovery behavior.

## 1. Introduction

Metal oxides have attracted the attention of many users and scientists interested in gas sensing under atmospheric conditions [1,2]. As an n-type semiconductor with a stable wide-band gap, SnO_2_ has received considerable attention in many application areas, such as gas sensors [3,4], solar cells [5], and lithium-ion batteries [6–8]. Nanostructured SnO_2_ films and electrospun granular hollow SnO_2_ nanofibers exhibit good sensitivity to hydrogen [9,10] at low temperatures. Wide investigation shows that nanostructures of SnO_2_ show high sensitivity to acetone gas, with short response times and fast recovery speeds [11–13]. Many methods have been focused on the synthesis of SnO_2_ nanostructure materials with different morphologies to improve the sensor performance. However, the method of producing the sensing film can change the porosity of the SnO_2_ nanomaterials, which influences the performance of sensors [14].

Molecular imprinting technology is considered to have considerable potential for use in recognition in various sensor applications [15–17]. Template molecules connect functional monomers and then these monomer-template adducts are polymerized. After the removal of the template from the polymer, the complementary binding sites are capable of template molecule recognition [18–20].

Herein, we designed highly sensitive SnO_2_-based acetone gas sensors enhanced by molecular imprinting technology. Acetone solutions were introduced during the synthesis of nanomaterials or/and device fabrication to produce appropriate structures which are more suitable for the adsorption and desorption of acetone gas. The as-synthesized SnO_2_ sensors were exposed to acetone gas with various concentrations ranging from 50 ppb to 100 ppm. The sensor produced by incorporating acetone both during the nanomaterial synthesis and device fabrication exhibits the best performance in terms of high sensitivity, fast response recovery and excellent repeatability.

## 2. Experimental Section

### 2.1. Synthesis of SnO_2_ Nanomaterials

#### 2.1.1. Synthesis of Unassisted SnO_2_ Nanomaterial W

Tin (II) chloride (SnCl_2_•2H_2_O, 1.5 g) was dissolved in 70 mL of water. Next 0.5 mL of hydrochloric acid (38%) was dropped into the mixture. The mixture was stirred for about 2 h at room temperature. The mixture solution was transferred into Teflon-lined stainless steel autoclaves, sealed tightly, heated and maintained at 90 °C for 20 h. The product was centrifuged, washed with water, and dried at 60 °C. Finally, SnO_2_ nanomaterial was obtained by annealing the product at 350 °C for 2 h.

#### 2.1.2. Synthesis of Acetone-Assisted SnO_2_ Nanomaterial A

Tin (II) chloride (SnCl_2_•2H_2_O, 1.5 g), 0.5 mL of hydrochloric acid (38%) and 10 mL acetone were dissolved in 60 mL of water. Then, the mixture was stirred for about 2 h at room temperature. After that, the mixture solution was transferred into Teflon-lined stainless steel autoclaves, sealed tightly, heated and maintained at 90 °C for 20 h. The product was centrifuged, washed with water, and dried at 60 °C. Finally, SnO_2_ nanomaterial was obtained by annealing the product at 350 °C for 2 h.

### 2.2. Device Fabrication

The as-prepared SnO_2_ powder was mixed with water/acetone to form a paste, then the paste was coated onto a ceramic tube (axial length 4 mm and basal diameter 1.5 mm) to form a thick film which has the thickness of about 100 μm. The ceramic tube was equipped with two pairs of Au electrodes and a Ni–Cr heater through the tube to control the experimental temperature. Table 1 shows that the four-type samples of films (WW, WA, AW and AA) were fabricated by mixing different as-prepared SnO_2_ nanomaterials with water/acetone. For example, AA was acquired by mixing SnO_2_ powder A with acetone and the gas sensor S_AA_ was fabricated by coating AA onto a ceramic tube.

### 2.3. Characterization and Measurement of Gas-Sensing Properties

The products were characterized by scanning electron microscopy (SEM, JSM-6700F; 5 kV, JEOL, Tokyo, Japan). X-ray power diffraction (XRD, D8 Avance, Brucker, Karlsruhe, Germany, λ = 1.5418) analysis was conducted to characterize the products in the range of 20°–80°. The porous structure has been further confirmed by the Nitrogen adsorption–desorption analysis (Belsorp Mini, Ankersmid, The Netherland). ^17^O-NMR (Nuclear Magnetic Resonance) was introduced to analysis the mechanism (AVANCE III 600, Bruker, Karlsuhe, Germany)

A calculated amount of acetone gas is introduced into a 10 L chamber by a syringe. A fan was fixed in the chamber to homogenize the acetone gas. The gas sensing properties were detected by a computer-controlled Navigation 4000-NMDOG gas-sensing measurement system (Zhongke nano IOT, Beijing, China). The gas response of the sensor was defined as R_a_/R_g_, where R_a_ and R_g_ are the resistance of gas sensors in air and acetone gas, respectively.

## 3. Results and Discussion

### 3.1. Structures of W/A and Films

**Figure 1.**
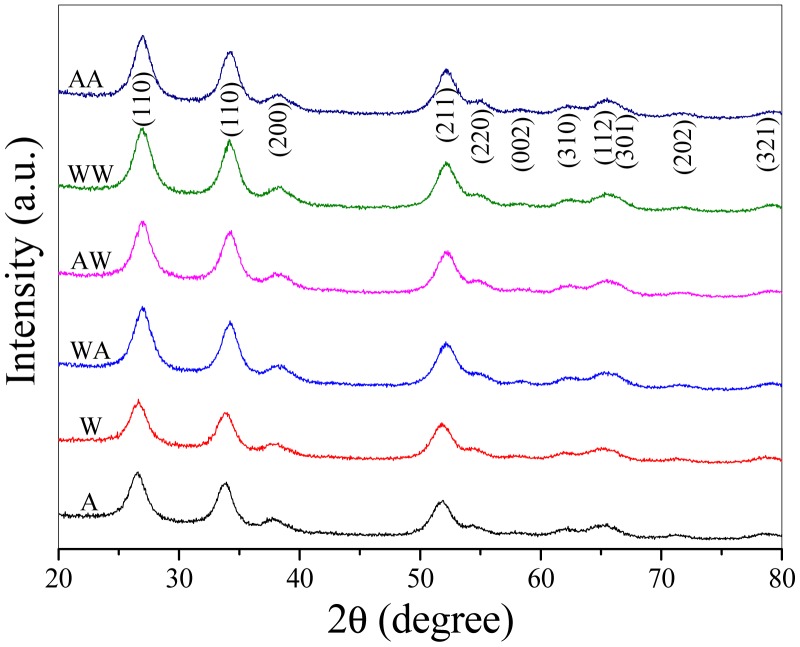
XRD patterns of as-prepared SnO_2_ nanomaterials and films.

**Figure 2.**
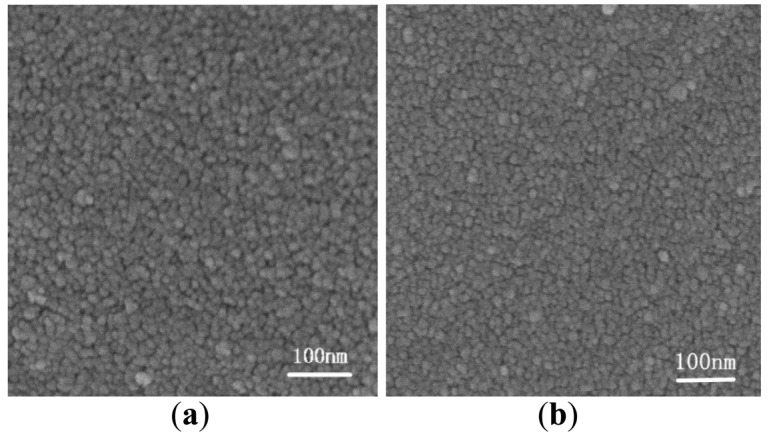
SEM images of (**a**) W: unassisted SnO_2_ nanomaterial; (**b**) A: acetone-assisted SnO_2_ nanomaterial.

The as-prepared SnO_2_ nonamaterials and the four-type films were examined by XRD. Figure 1 shows all the distinguishable peaks were well indexed to the tetragonal rutile structure of cassiterite SnO_2_ (JCPDS file No. 41-1445) [21]. There were no characteristic peaks of any other impurities. Figure 2 shows the uniform particle size of W and A. It can be found that A is characterized by more elaborate particle structure.

**Figure 3.**
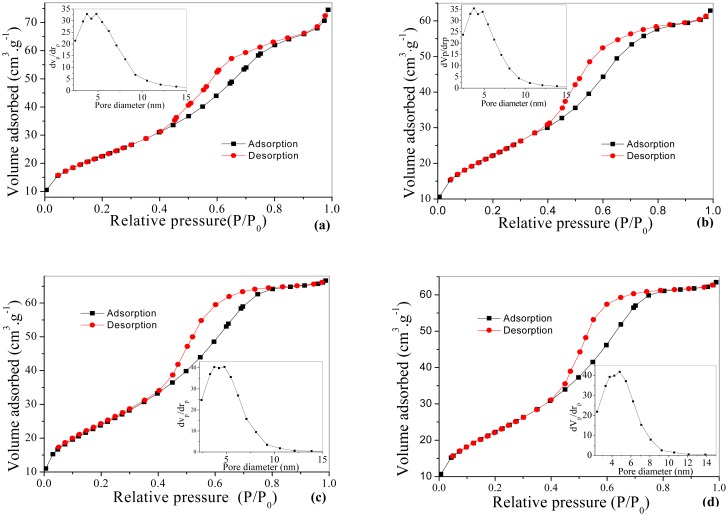
Nitrogen gas adsorption-desorption isotherms and BJH pore size distribution (inset) of (**a**) W; (**b**) A; (**c**) WW; (**d**) AA.

The porosities of nanoparticles are distinctly confirmed by N_2_ adsorption–desorption analysis [22]. Figure 3a,b show that W and A have the adsorption-desorption isotherm shape of IV with the type H1 hysteresis loop, which is usually attributed to the thermodynamic or/and network effects. Figure 3c,d show that the productions of pastes WW and AA exhibit pure type IV and the hysteresis loops transform form H1 to H2. The presence of the hysteresis loops is indicative of the pores in a 3D intersection network [23,24]. Insets of Figure 3a,b show the Barrett-Joyner-Halenda (BJH) pore size distributions of as-prepared materials W and A with the average pore size of about 5.53 and 4.68 nm (see Table 2) respectively. Table 1 lists the average pore size of WW, WA, AW and AA about 4.61, 4.33, 4.79 and 4.72 nm. We see that the change of hysteresis loops, pore size distributions and Brunauer-Emmett-Teller surface areas (see Tables 1 and 2) means the procedure of device fabrication has effect on the structure of as-prepared materials, which result in the change of sensing performance of sensors.

**Figure 4.**
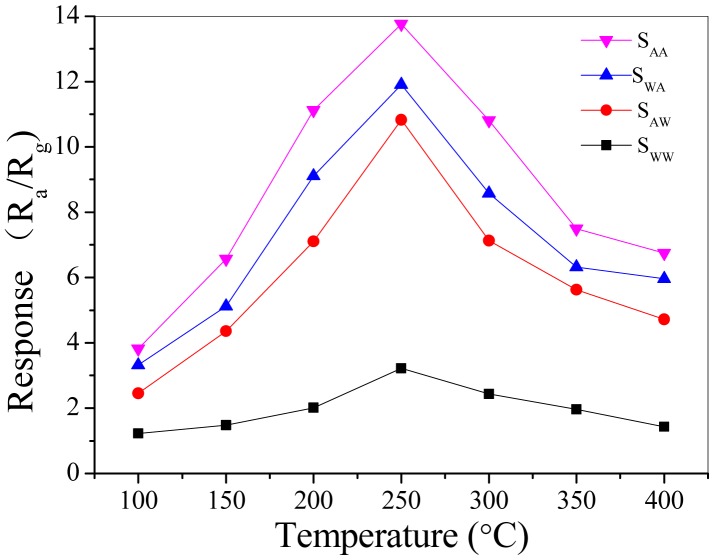
Response (R_a_/R_g_) for different samples to 50 ppm acetone gas at various operating temperatures.

The response measurements to 50 ppm acetone gas for the different sensors were performed from 100 °C to 400 °C. Figure 4 shows all the gas sensors possess the best response performance at the operating temperature of 250 °C. Thus, 250 °C is chosen as the optimized operating temperature for acetone gas sensing study [25,26].

**Figure 5.**
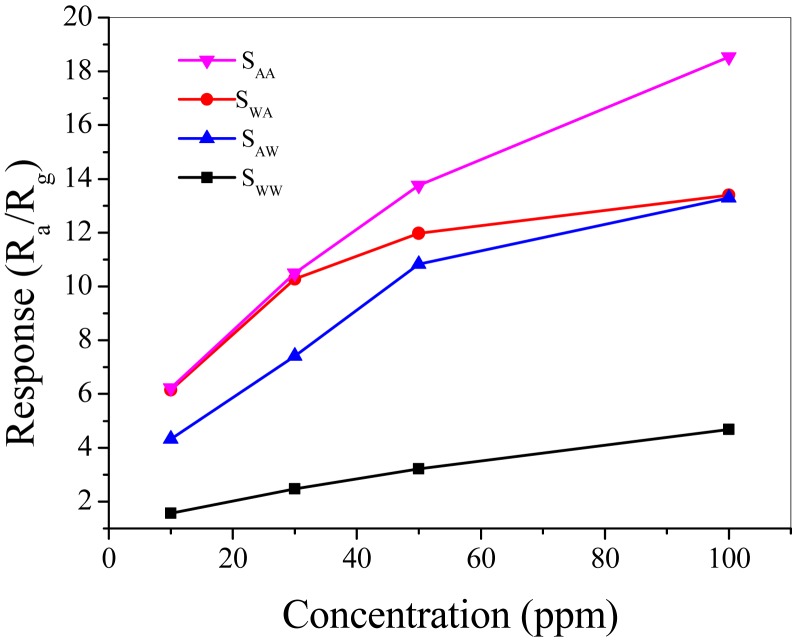
Response of sensors at 250 °C *versus* acetone concentrations.

From Figure 5 we see that the response of sensors increased with the increase of the acetone concentration at 250 °C. The response descended in the order of S_AA_, S_WA_, S_AW_ and S_WW_. The response of sensor S_AA_ was about 18.5 to 100 ppm acetone. When the concentration of acetone was low, S_AA_ and S_WA_ exhibit higher response compared with S_AW_ and S_WW_. When the acetone concentrations reach higher levels, the response of S_WA_ is close to that of S_AW_. The response of S_WW_, S_AW_ and S_WA_ gradually tended to saturation compared with S_AA_ with the increase of acetone concentrations.

**Figure 6.**
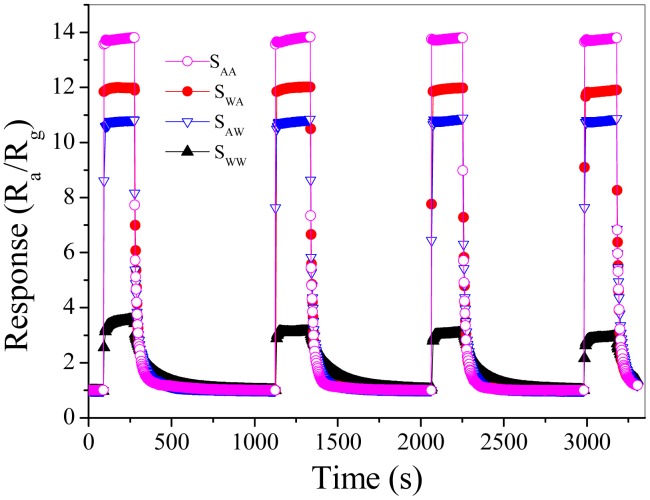
Responses to 50 ppm acetone gas for S_AA_, S_WA_, S_AW_ and S_WW_ at 250 °C.

**Figure 7.**
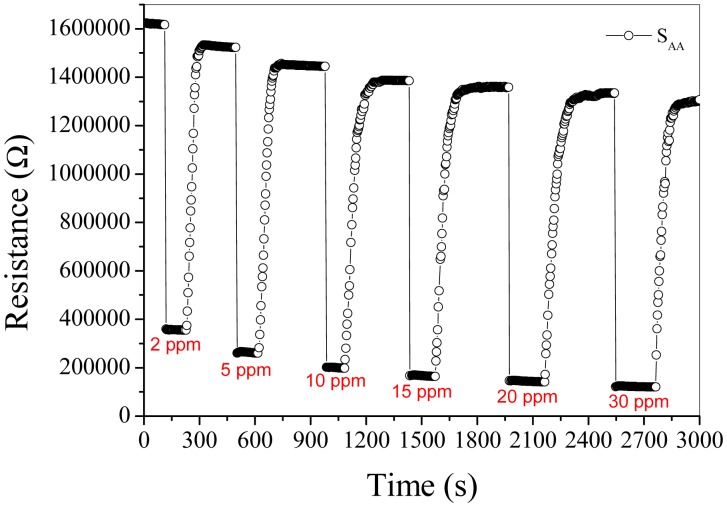
Response transients to different concentrations of acetone for S_AA_ at 250 °C.

**Figure 8.**
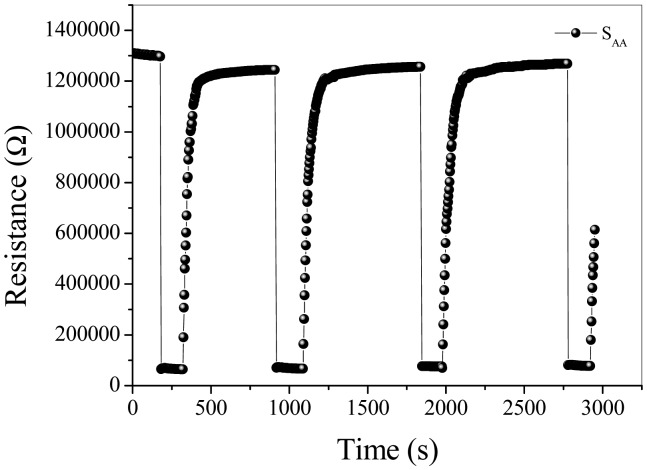
Repetitive response and recovery of S_AA_ to 100 ppm acetone gas at 250 °C.

**Figure 9.**
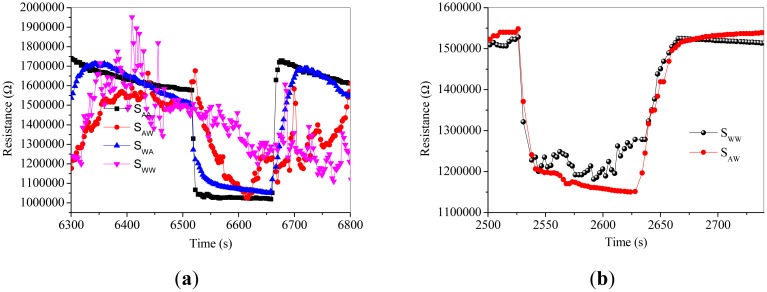
(**a**) Response of the four types of sensors to 50 ppb acetone at 250 °C; (**b**) Response of S_WW_ and S_AW_ to 300 ppb acetone at 250 °C.

Figure 6 shows the dynamic response of sensors to 50 ppm acetone at the operating temperature of 250 °C. It is obvious that the response of S_AA_, S_WA_, S_AW_ and S_WW_ to 50 ppm acetone is about 13.8, 11.9, 10.8 and 3.2, respectively, and the response/recovery speed descends in the order of S_AA_, S_WA_, S_AW_ and S_WW_. Figure 7 shows the baseline drift [14,27,28] when S_AA_ is orderly exposed to different low concentrations of acetone at 250 °C. It should be noticed that R_a_ of S_AA_ does not decrease sharply when the acetone concentrations are above 15 ppm. As shown in Figure 8, the reversible cycles of the response and recovery curve for S_AA_ indicate the stable and repeatable characteristic when the surrounding environment is cycled between clean air and 100 ppm acetone. Figures 7 and 8 tell us that the baseline drift is merely relevant to gas concentrations. Figure 9 shows different sensing behaviors of the four-type sensors to low concentrations of acetone gas. Figure 9a shows the detection limits [29,30] of S_AA_ and S_WA_ are is low as 50 ppb and from Figure 9b we can see that S_AW_ and S_WW_ have detection limits of about 300 ppb.

Table 3 shows the response time of S_WW_, S_AW_, S_WA_ and S_AA_ about 9.2–22.1 s, 8.2–17.3 s, 7.1–13.8 s and 4.3–6.7 s (defined as the time needed to reach 90% of total signal change) at various acetone gas concentrations, respectively. It is obviously recovery times descend in order of S_WW_, S_AW_, S_WA_ and S_AA_.

### 3.2. Analysis of Gas Sensing Mechanism

**Figure 10.**
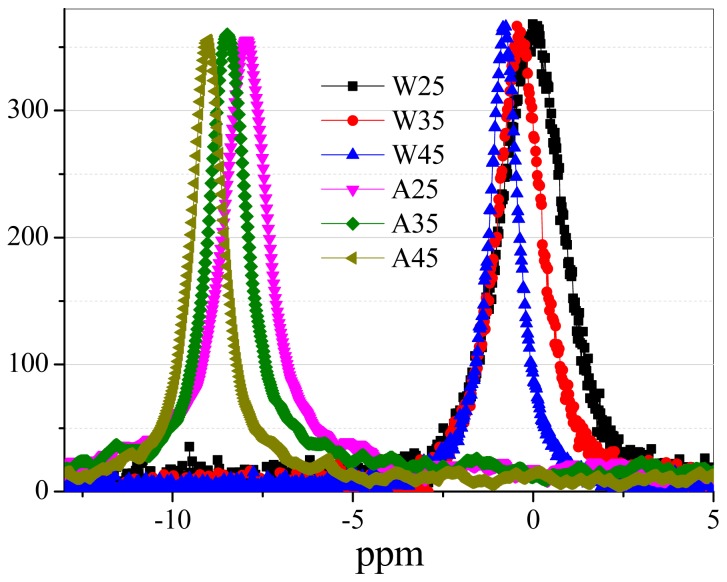
^17^O-NMR spectroscopy of water and acetone. W25, W35, W45, A25, A35 and A45 represent the ^17^O-NMR spectra of water and acetone, respectively, at 25, 35 and 45 °C.

**Figure 11.**
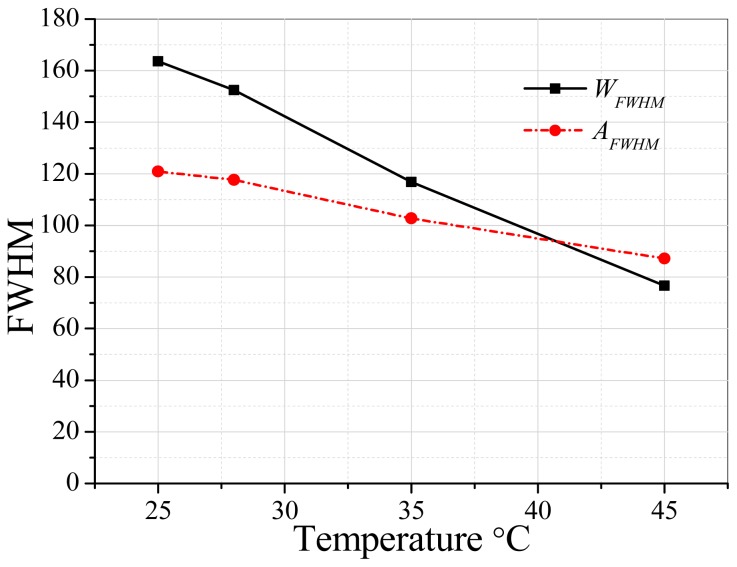
FWHM of water and acetone *versus* temperature.

**Figure 12.**
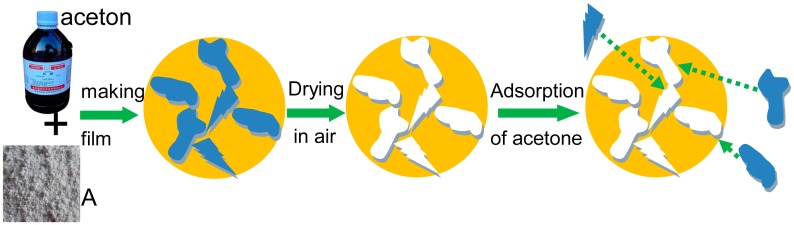
Schematic illustration of imprinting by acetone clusters on acetone-assisted SnO_2_ film.

S_AA_ displaying good gas-sensing performance can be due to two possible mechanisms. First, the liquid of target gas (acetone) is introduced during the procedure of SnO_2_ nanomaterial synthesis to optimize the structure of SnO_2_ nanomaterial for the smooth adsorption and desorption of acetone gas. We noticed that S_AW_ exhibits a higher response compared with S_WW_ and S_AA_ has excellent performance in contrast to S_AW_, which means that acetone-assisted nanomaterial A has an excellent structure for acetone gas sensing in contrast to W. Table 1 shows that the nanomaterial A has the average pore size of about 4.68 nm which is lower than that of W (about 5.53 nm), which may be the key factor for improving the sensors' performance. The relatively low BJH of A can be due to the small acetone clusters [31] in contrast to water clusters [32,33]. Water has more chance to be in larger size clusters thanks to its abundant hydrogen bonds. Figure 10 shows the ^17^O-NMR spectra [34] of water and acetone at 25, 35 and 45 °C based on a Bruker AVANCE III 600 instrument. Figure 11 shows that the ^17^O-NMR spectroscopy full width at half maximum intensity (FWHM) of water almost has a linear decrease trend and it reduces much faster than that of acetone with the rise of temperature. It further confirms that water has abundant hydrogen bonds, which can result in large sized clusters of water in contrast to acetone. For the second possible mechanisms, the method of device fabrication plays very import role in enhancing the response of gas sensors. The representative schematic mechanism for the device fabrication can be illustrated as in Figure 12. Acetone-assisted SnO_2_ is mixed with acetone solution to form a paste, and then this is coated onto a tube to form a film where acetone clusters can interact with SnO_2_. After drying in air, the acetone-assisted SnO_2_ nanomaterial is imprinted by acetone clusters. The memorized structure is suitable for the adsorption and rapid diffusion of acetone clusters when S_AA_ was exposed to acetone gas and clear air alternatively during the tests to finally improve the sensing capability.

## 4. Conclusions

In conclusion, we have developed a novel method for designing highly sensitive acetone gas sensors enhanced by molecular imprinting. In this work, we not only have focused on the synthesis of SnO_2_ nanomaterials, but also emphasized the device fabrication method. Thanks to the introduction of acetone both in the synthesis and device fabrication phases, the sensor S_AA_ exhibits the best sensitivity with the highest response and fastest response/recovery speed. Compared with S_AW_ and S_WW_, the sensing performance of S_AA_ and S_WA_ exhibited improved responses to acetone. It reveals that the procedure of device fabrication plays an essential role in the design of sensors. Our design method proved to be a new route to devise ultra-sensitive acetone sensors, which may be instructive for the design of special high sensitivity gas sensors.

## Figures and Tables

**Table 1.** Average pore size and BET surface area of four-type films.

**Sensors**	**Film**	**Materials**	**Liquid**	**Average Pore Size (nm)**	**BET (m^2^ g^−1^)**
S_WW_	WW	W	water	4.6125	89.351
S_WA_	WA	W	acetone	4.3275	92.703
S_AW_	AW	A	water	4.7853	80.901
S_AA_	AA	A	acetone	4.7227	83.079

**Table 2.** Average pore size and BET surface area of SnO_2_ nanomaterials.

**Materials**	**Acetone:Water**	**Average Pore Size (nm)**	**BET Surface Area (m^2^ g^−1^)**
W	0:70	5.5312	83.35
A	10:60	4.6811	83.05

**Table 3.** Response speed of different sensors under various acetone gas concentrations.

**Concentration of Gas (ppm)**	**Response Time (s)**				**Recovery Time (s)**			
	
	**S_WW_**	**S_AW_**	**S_WA_**	**S_AA_**	**S_WW_**	**S_AW_**	**S_WA_**	**S_AA_**
10	22.1	17.3	13.8	6.7	S_WW_	142.1	69.2	68.5
30	13.3	9.8	8.7	5.3	292.6	156.3	85.9	78.1
50	9.7	8.7	7.7	5.0	456.2	144.6	133.4	119.8
100	9.2	8.2	7.1	4.3	463.5	231.1	212.3	156.3
